# IRAK4 inhibition: an effective strategy for immunomodulating peri-implant osseointegration via reciprocally-shifted polarization in the monocyte-macrophage lineage cells

**DOI:** 10.1186/s12903-023-03011-0

**Published:** 2023-05-08

**Authors:** Juan Zhao, Jia Li, Antian Xu, Yangbo Xu, Fuming He, Yingjie Mao

**Affiliations:** 1 Department of ProsthodonticsSchool of StomatologyZhejiang Provincial Clinical Research Center for Oral Diseases, Stomatology HospitalZhejiang University School of MedicineKey Laboratory of Oral Biomedical Research of Zhejiang Province, Cancer Center of Zhejiang University, Hangzhou, 31000 China; 2 Department of Prosthodontics, The Affiliated Stomatology Hospital, Zhejiang University School of Medicine, 166 QiuTao Rd(N), Hangzhou, 310000 China

**Keywords:** Implant, IRAK4, Macrophage, Osteoclast, Foreign body giant cell, Osseointegration

## Abstract

**Background:**

The biomaterial integration depends on its interaction with the host immune system. Monocyte-macrophage lineage cells are immediately recruited to the implant site, polarized into different phenotypes, and fused into multinucleated cells, thus playing roles in tissue regeneration. IL-1R-associated kinase 4 (IRAK4) inhibition was reported to antagonize inflammatory osteolysis and regulate osteoclasts and foreign body giant cells (FBGCs), which may be a potential target in implant osseointegration.

**Methods:**

In *in-vitro* experiments, we established simulated physiological and inflammatory circumstances in which bone-marrow-derived macrophages were cultured on sand-blasted and acid-etched (SLA) titanium surfaces to evaluate the induced macrophage polarization, multinucleated cells formation, and biological behaviors in the presence or absence of IRAK4i. Then, bone marrow stromal stem cells (BMSCs) were cultured in the conditioned media collected from the aforementioned induced osteoclasts or FBGCs cultures to clarify the indirect coupling effect of multinucleated cells on BMSCs. We further established a rat implantation model, which integrates IRAK4i treatment with implant placement, to verify the positive effect of IRAK4 inhibition on the macrophage polarization, osteoclast differentiation, and ultimately the early peri-implant osseointegration in vivo.

**Results:**

Under inflammatory conditions, by transforming the monocyte-macrophage lineage cells from M1 to M2, IRAK4i treatment could down-regulate the formation and activity of osteoclast and relieve the inhibition of FBGC generation, thus promoting osteogenic differentiation in BMSCs and improve the osseointegration.

**Conclusion:**

This study may improve our understanding of the function of multinucleated cells and offer IRAK4i as a therapeutic strategy to improve early implant osseointegration and help to eliminate the initial implant failure.

**Supplementary Information:**

The online version contains supplementary material available at 10.1186/s12903-023-03011-0.

## Introduction

Biomedical device implantation is always accompanied by inflammation, both through the damage caused by the surgical procedure and the biomaterial itself. The biomaterial integration depends on its interaction with the host immune system, in which immune cells are recruited to the damaged sit and then a cascade of cellular and molecular events is initiated [1, 2]. Even if nowadays most dental implants work well over long-term follow-up, there are still chances for implant failures with inexplicable reasons. One explanation for osteointegration failure may be due to peri-implant immunological disbalance [3].

Biomaterials implantation as an inducer can activate neutrophils via toll-like receptors (TLRs) and prepare an immunological microenvironment. Activated neutrophils will release macrophage inflammatory protein-1α (MIP-1α), MIP-1β and interferon-γ (IFN-γ) to recruit more macrophages [4]. After being recruited to the "battlefield", macrophages begin to clear cell debris and other metabolites through phagocytosis, and then will be polarized into different phenotypes, thus playing different roles [5]. During this period, macrophages become the more dominant cell type and recruit other immunocytes to join the tissue repair process, which is crucial for inflammation resolution and tissue regeneration [6].

During the initial phase after implantation, proinflammatory macrophages induce acute responses to trauma and foreign bodies, whereas anti-inflammatory macrophages modulate the resolution of inflammation and induce the following healing stage [7, 8]. Moreover, macrophages can fuse to multinucleated cells under a complex network of cytokines and molecules in the osteoimmune microenvironment [9]. Multinucleated cells were initially characterized as ‘bad’ foreign body cells or the end-point of rejection or fibrosis of biomaterials [10, 11]. However, more recently, not only osteoclasts but also foreign body giant cells (FBGCs) have been observed around implant-bone interfaces even years after placement [12]. They could be found without obvious inflammatory reaction or fibrous encapsulation [13], indicating that the formation and existence of multinucleated cells may lead to bone-implant integration into a foreign body equilibrium [14]. Therefore, the biological functions of multinucleated cells and their interactions with osteogenic cells are tightly bound to bone healing [15, 16]. Some studies have shown the necessity to no longer refer to multinucleated cells as ‘‘good’’ or ‘‘bad’’ cells, but more scientifically describe them specifically as M1- multinucleated cells and M2-multinucleated cells [10]. As far as dental implants are concerned, the potential linkages and mechanisms underlying the interaction of induced macrophage polarization, subsequent multinucleated cells formation, and their role in implant osseointegration remain poorly understood.

Interleukin-1 receptor-activated kinase 4 (IRAK4) is a key regulator of the IL-1R and TLR-mediated signaling [17]. It binds the adaptor protein MyD88 to transmit signals from IL-1R and TLRs, the downstream result of which is the activation of nuclear factor kappa B (NF-κB), interferon regulatory factor 5 (IRF5), and mitogen-activated protein kinase (MAPK) pathways [18]. These signaling pathways are essential for the induction of osteoclast differentiation from mononuclear phagocytes [19].

Recently, several studies have highlighted IRAK4 may be an attractive target for treating inflammatory and autoimmune diseases [20, 21]. It showed that IRAK4 could antagonize inflammatory osteolysis, and IRAK4 inhibitor (IRAK4i) therapy attenuated rheumatoid arthritis (RA) disease activity by blocking M1 macrophage activation and also disrupted osteoclastogenesis [22]. Loss of IRAK4 inhibit stimulated osteoclastogenesis under inflammatory conditions without altering physiological bone resorption. Furthermore, IRAK4 deficiency up-regulated the M2-related gene expression and restored the formation of FBGC after in vitro treatment of inflammatory factors [23]. However, the role of IRAK4 and its effect on osteoclast and FBGC formation in bone implantation models were not clear. Whether IRAK4i could act as a potential therapeutic target in preventing excessive bone resorption in acute inflammatory or infectious situation after implantation via regulating the polarization of macrophages and the balance between osteoclasts and FBGCs are worth exploring.

Regarding the potential effect of IRAK4 in osseointegration, we hypothesize that IRAK4 plays a pivotal role in bone/biomaterial homeostasis. That is, it can regulate the polarization of macrophages and the formation of multinucleated giant cells. Further, its potential regulation of osteoclasts and FBGCs can result a shift in their biological functions, and via cytokines secretion, it also has the potency to stimulate the osteogenic differentiation of bone marrow stromal stem cells (BMSCs) indirectly.

To justify the hypothesis, we established simulated physiological and inflammatory circumstances in which bone-marrow-derived macrophages (BMMs) were cultured on sand-blasted and acid-etched (SLA) titanium surfaces to evaluate the induced macrophage polarization, multinucleated cells formation, and biological behaviors in the presence or absence of IRAK4i. Then we cultured BMSCs in the conditioned media collected from the aforementioned induced osteoclasts or FBGCs cultures to clarify the indirect coupling effect of multinucleated cells on BMSCs in vitro. We further established a rat implantation model, which integrates IRAK4i treatment with titanium implant placement, to verify the positive effect of IRAK4 inhibition on the macrophage polarization, osteoclast differentiation, and ultimately the early peri-implant osseointegration in vivo.

## Methods

### Titanium sample preparation

The commercial pure titanium disks (10 mm in length, 1 mm in thickness for in vitro experiments) and screw-shaped titanium implants (Ø 2 × 4 mm for in vivo ones) were prepared. The sandblasted and double-acid etched titanium (SLA) surface was prepared as previously described [24]. Before the following procedures, all samples were sterilized by UV irradiation.

### Isolation and culture of BMMs

BMMs were obtained from the femora of 6-week-old male C57/BL6 mice. Briefly, bone marrow of the femoral midshaft was flushed out and suspended in alpha minimum essential medium (α-MEM, HyClone, USA) supplemented with 10% fetal bovine serum (FBS, HyClone) and 100 U/ml of penicillin and streptomycin. The cells were centrifuged and then resuspended in a red blood cell lysis buffer (Solarbio, China) for 5 min on ice. After centrifugation, the sedimentary cells were resuspended and non-adherent cells were collected after 24 h in α-MEM containing 30 ng/ml macrophage colony-stimulating factor (M-CSF, R&D System, USA) which was involved in the commitment of the monocyte/macrophage lineage and participates in the induction of osteoclasts.

### Cell viability assay

BMMs were inoculated on a 24-well plate and cultured in α-MEM containing 30 ng/ml M-CSF and various concentrations (0, 15.625, 31.25, 62.5, 125, 250, 500 or 1000 nM) of IRAK4i (BMS-986142, Selleck, China). After culturing for 1, 3, 5 days, the medium was replaced with α-MEM complemented with 10% (v/v) AlamarBlue reagent (Invitrogen, Grand Island, NY). A microplate reader (SpectraMax iD3, USA) was used to evaluate cell viability at a wavelength (λex = 540 nm, λem = 590 nm). The results were analyzed by normalizing optical density against cell density.

### Macrophage polarization

#### Immunological staining of macrophages

BMMs were cultured on various titanium disks in α-MEM with 30 ng/ml M-CSF for 24 h. Then cells were fixed with 4% (v/v) paraformaldehyde (PFA, Solarbio) for 15 min and rinsed with PBS. After permeabilized with 0.5% Triton X-100 and blocked by 1% bovine serum albumin (BSA), the disks were incubated with CCR7 antibodies (GTX19079, GeneTex, USA) for M1 phenotype and CD206 antibodies (sc-365938, Santa, USA) for M2 phenotype at 4 ℃ overnight. Alexa Fluor 488-conjugated goat anti-rabbit secondary antibodies (ab150113, Abcam, UK) were used for the detection. Cell nuclei were counterstained with 4′,6-diamidino-2-phenylindole dihydrochloride (DAPI). Cells were visualized by a laser confocal scanning microscope (LCSM, Nikon A1, Japan).

### Real-Time qPCR

For RT-qPCR, BMMs were seeded on the SLA surfaces placed in a 24-well plate and cultured in α-MEM containing 30 ng/ml M-CSF with or without IL-1β and IRAK4i for 3 days. Total RNA of cells was isolated and purified by a TRIzol® reagent (AmbionTM, Life Technologies Pty Ltd, Australia). The mRNA relative expression levels of indicated genes were detected by a SYBR Green I kit (Takara, Osaka, Japan) via the Applied Biosystems ViiA 7 (Thermo Fisher Scientific, USA). All primer sequences are listed in Table 1. Each sample was analyzed in triplicate, and the expression level of target genes is normalized to the housekeeping gene *actin* as a control using the comparative Ct (2^−ΔΔCT^) method.

**Table 1:** Primer used for real-time PCR

Gene	Forward primer sequence (5’-3’)	Reserve primer sequence (3’-5’)
Actin	TGGAATCCTGTGGCATCCATGA	TAAAACGCAGCTCAGTAACAGT
iNOS	GACGAGACGGATAGGCAGAG	CACATGCAAGGAAGGGAACT
Il-6	ACCCCAATTTCCAATGCTCT	CGCACTAGGTTTGCCGAGTA
Tnf-α	CACAGCCTTCCTCACAGAGC	CCCGGCCTTCCAAATAAATA
Il-10	CCTGCTCTTACTGACTGGCATGAG	TGAAGGCAGTCCGCAGCTCTAG
Tgf-β	GACCTGGGTTGGAAGTGGAT	TTGGTTGTAGAGGGCAAGGA
Bmp2	CGCTCCACAAACGAGAAAAG	GTCATTCCACCCCACATCAC
Trap	CACGATGCCAGCGACAAGAGG	ATCTGTGCAGAGACGTTGCCAAG
Ctsk	GGAGGCCTCTCTTGGTGTCC	TCGATGGAGTCTGGGACCCT
Sclerostin	CGTGCCTCATCTGCCTACTTGTG	CCGGTTCATGGTCTGGTTGTTCTC
Sema 4D	CTACAGCCACTAAGCAGCACTTCC	GTCAAGTTCAGGTGGTCACAGGTG
Cthrc1	GCTACAGTTGTCCGCACCGATC	GCCACTGAACAGAACTCGCAGAG
Bmp6	GCTCCGGTTCTTCAGACTACAACG	GCAATGATCCAGTCCTGCCATCC
Mrc1	ACCTGGCAAGTATCCACAGCATTG	TGTTGTTCTCATGGCTTGGCTCTC
Ym1	GAATGAAGGAGCCACTGAGGTCTG	TTGTTGTCCTTGAGCCACTGAGC
Alox15	CTCAGGCTTGCCACTTCATCACC	CGTCGCCATCAGCTTCTCCATC
Hla-dr	TGTCTCTGTCCTGGTGGCTCTG	TTCATGCGAAGGCTCTCCAGTTG

### Western blotting

Cells were then lysed with radio-immunoprecipitation (RIPA) lysis buffer and then collected after 15 min of centrifugation at 12,000 rpm at 4 ℃. The protein concentrations were measured by a BCA Protein Assay Kit (Takara, Osaka, Japan). The equivalent amount of protein samples was loaded onto SDS-PAGE and transferred to a PVDF membrane (Millipore Corp.). After blocking with 5% skim milk for 2 h, separated samples were incubated with the primary IRAK4 (DF2667, Affinity, USA), iNOS (13,120, CST, USA), CCR7, CD206 or CD163 (BSM54105, Bioss, China) antibodies at 4 ℃ overnight. The immunoreactive bands were detected using an ECL chemiluminescence reagent (AlphaEaseFC, USA) and visualized using a chemiluminescence imager (Bio-Rad, USA). Part of the blots was cropped, since the membrane was cut before antibody incubation to save the antibodies, and during the preparation of this manuscript, the blots of a smooth titanium disk group was deleted to conform to the topic of this study clearly and concisely.

### Inductive osteoclast and FBGC cultures in vitro

BMMs were cultured with 30 ng/ml M-CSF for 3 days. The cells were seeded on the titanium disks placed in a 24-well plate. In order to induce osteoclasts, the medium containing 50 ng/ml M-CSF (R&D System) and 50 ng/ml RANKL (R&D System) was used and changed every other day. For inducing FBGC, 50 ng/ml GM-CSF (Peprotech Ltd, USA) and 50 ng/ml IL-4 (Peprotech Ltd) was used in the medium that changed every other day. After another 4–5 days, osteoclast and FBGC differentiation and maturation were observed by microscopy.

### Osteoclast and FBGC formation

To verify the effect of IRAK4i on osteoclast and FBGC formation in simulated inflammatory microenvironment in vitro, the cells were cultured in osteoclast and FBGC-inductive mediums with or without the supplement of 50 nM IRAK4i or 15 ng/ml IL-1β.

### Cell identification

The osteoclasts and FBGCs were fixed with 4% (v/v) PFA for 15 min. The cells were stained with TRAP (Acid Phosphatase kit, Sigma) or May-Grünwald Giemsa (MGG Kit, Solarbio) kits, respectively, and observed under a microscope (CKX41, Olympus, Japan).

For TRAP staining, the osteoclasts were fixed with 3% PFA-2% sucrose for 30 min, permeabilized with Triton 0.5% in HEPES for 5 min and stained using naphthol AS-BI phosphoric acid and tartrate solution for 60 min at 37 °C. TRAP^+^ with 3 or more than 3 nuclei were counted as osteoclasts.

For May-Grünwald and Giemsa staining, the FBGCs were fixed with 4% PFA for 15 min. The cells were then successively stained with MGG working solution according to the manufacturer’s instruction for 10 min. After washed with ddH_2_O twice, MGG^+^ cells with 3 or more than 3 nuclei were counted as FBGCs.

### *Cell morphology *via* IF*

To observe the morphology of cells, the cells were fixed with 4% PFA for 20 min. After washing with PBS, cells were permeabilized with 0.5% Triton X-100, followed by incubated with TRAP antibody (GTX19079, GeneTex) and HLA-DR antibody (sc-515785, Santa) respectively. Cytoskeleton and cell nuclei were labeled with rhodamine-phalloidin (Cytoskeleton, Inc) and DAPI. The cell morphology was observed and photographed under LCSM.

### RT-qPCR

See above description about RT-qPCR for the subsequent steps.

### Western blot analysis

Protein expression levels of the osteoclast and FBGC was evaluated by Western blot with the TRAP, CTSK (ab207086, Abcam), c-Fos (ab222699, Abcam), and HLA-DR antibodies. See above description about western blotting for the subsequent steps.

### Osteogenic differentiation of BMSCs in conditioned medium

#### Rat BMSCs culturing

BMSCs were isolated from 3–4 weeks old Sprague–Dawley (SD) rats. The cells were cultured in the α-MEM at 37 °C with 5% CO_2_. CM was collected from the primary mouse osteoclasts and FBGCs cultured on different titanium disks as described in 2.6. To assess the effect of CM on osteogenic differentiation of BMSCs, cells were seeded in a 24-well plate with a mixture of the CM and an OM (50% v/v). The OM was α-MEM supplemented with 10 nM dexamethasone, 100 μM ascorbic acid, and 10 mM β-glycerophosphate. The mixture of medium was changed twice per week.

### Alkaline phosphatase activity

After 4 and 7 days, BMSCs were lysed with Triton X-100. The supernatant was collected after centrifugation of 12,000 rpm/min at 4 °C for 15 min. The level of ALP activity was measured using a LabAssayTM ALP Assay Kit (Wako, Osaka, Japan) and was normalized to a total amount of cellular protein.

### Alizarin red staining and quantitative assessment of calcium nodules

After 21 days, cell layers were washed by PBS and then fixed with 4% PFA for 15 min, and then stained with 0.2% alizarin red solution (Solarbio) for 20 min. Staining cells were washed and mineralized nodules were photographed under a light microscope. For quantitative analysis of alizarin red of cell layers, 1 ml of 10% cetylpyridinium chloride (C129534, Aladdin) was added into each well. After the dissolution of calcium nodules, 200 μl solution of each well was transferred to a 96-well plate. The optical density was detected by a microplate reader at the wavelength of 590 nm.

### Animals and surgery procedures

Seventy-two male SD rats about 300 g in weight were used. The rats were housed under 25℃ with a 12 h light/dark cycle and were allowed free access to food and water. One-half of the SD rats were injected with IRAK4i (2500 nM, 100 μl) subperiosteally at the implantation sites for 3 consecutive days prior to the surgery. The expression of IRAK4 was verified by WB assay. The other half was injected with 100 μl PBS as control. Then, SD rats received an intraperitoneal injection of 10% chloral hydrate (0.33 ml/100 g, Aladdin) and local injection of 2% lidocaine (Shiyao Yinhu company, China) before implantation. After shaving and disinfecting the implantation areas, the soft tissue of the tibiae was dissected and the flaps were elevated to expose the underlying bone tissue. A hole was drilled with a carbide bur at a slow rotary speed under profuse irrigation with 0.9% NaCl. SLA implants were screwed into the left and right tibias of rats, respectively. The periosteum and skin were sutured postoperatively. Penicillin (400,000U/d) was injected intramuscularly into the rats instantly. After 3, 7, 28 days of submerge healing, a total of 72 rats were sacrificed with a lethal dose of anesthetic. Tissue specimens containing implants were collected for immunohistochemical and histomorphometric evaluation. All the experiments were approved by the Institutional Animal Care and Use Committee of Zhejiang University, Hangzhou, China (ZJU20190610).

### Histomorphometric assessment

To observe the new bone formation, the proximal tibiae containing the implants were taken off and fixed in 10% buffered formalin (Solarbio) for 3 days. Specimens were successively dehydrated with 75%, 85%, 95%, 100% alcohol prior to being embedded with polymethyl-methacrylate (PMMA). After polymerization, disks of 200 μm thickness were prepared along the longitudinal axis of the implant and then polished sequentially with sandpapers to a final thickness of 40 μm. The non-decalcified sections were then stained with methylene blue and acid fuchsin. A light microscope and an Image-Pro Plus were used to capture the images and to analyze the histological phenotypes. The BIC% was calculated as the linear percentage of direct bone-implant contact over the total implant interface in cancellous bone.

### Histomorphometry

For immunohistochemical staining, the tibiae were excised, and the surrounding soft tissue was cleaned off. After fixed in 4% PFA for 24 h and decalcified with 10% ethylene diamine tetraacetic acid (EDTA, Solarbio) for 3 weeks, the implants were screwed out counterclockwise. The decalcified bone blocks were dehydrated in graded series of ethanol, embedded in paraffin, and cut into 4 μm disks perpendicular to the long axis of the implants. Subsequently, bone disks were incubated with 3% hydrogen peroxide for 10 min and then placed in an autoclave at 121℃ for 15 min in sodium citrate buffer.

To identify M1 macrophage, M2 macrophage, osteoclasts and FBGCs, CCR7, CD206, TRAP, and HLA-DR were used as markers, respectively. After blocked with 0.5% BSA for 1 h, the disks were incubated with primary antibodies. For histological analysis of TRAP activity, bone disks were stained with 50 mM sodium tartrate and naphthol AS-TR phosphate (Sigma). For quantitative assessment, the area at a distance of 500 μm from the circular bone-implant interface was regarded as ROI, and 5 images per section within the ROI were randomly chosen and photographed by a microscope. The expressions levels of CCR7, CD163, and HLA-DR were indexed as the percentage of the positive staining area *vs.* the whole area per disk. Osteoclast numbers were counted according to TRAP^+^ and being of more than or equal to 3 nuclei.

### Statistical analysis

Data are expressed as mean ± SD, and each experiment was repeated at least 3 times. As for in vivo animal model, 6 rats per each time points and treatment group were included. The sample sizes were selected based on previous literatures. For qPCR, western blot, ALP, and mineralization quantification, data were analyzed for statistical significance by two-way ANOVA, as implemented in GraphPad Prism 6, and *p* < *0.05* was considered statistically significant. Scatter diagrams were made with GraphPad Prism 6, regression analyses were conducted, and the most accurate trend lines were displayed according to P value.

## Results

### The effect of IRAK4i on macrophage polarization cultured on the SLA surface in the presence of IL-1β

**Fig. 1:**
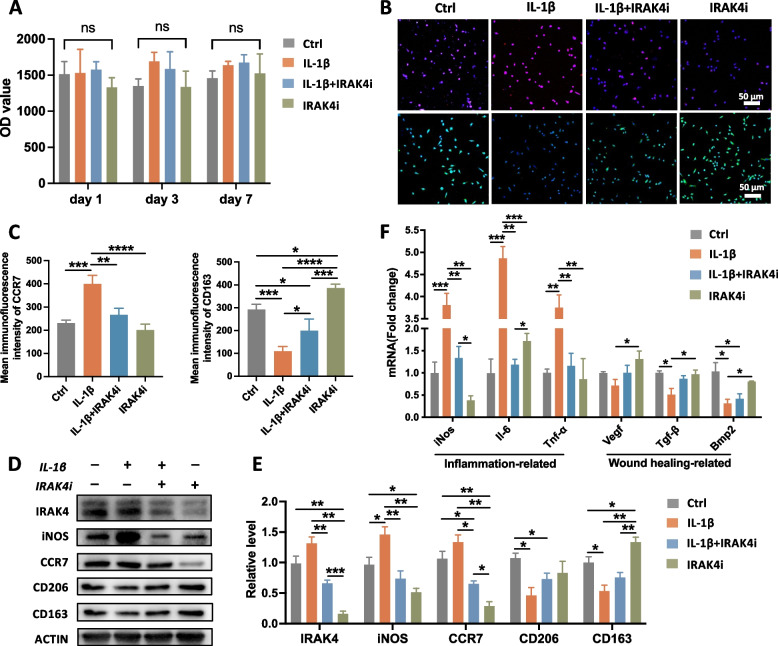
The polarization and differentiation of the bone monocyte/macrophage lineage cells on SLA surface under inflammatory conditions. **A** BMMs were cultured on the SLA surfaces in the presence of M-CSF (30 ng/ml) with or without IL-1β and IRAK4 inhibitor (IRAK4i) for 1, 3, 7 days, and then the cell viability was evaluated by AlamarBlue assay. **B** Representative images of immunofluorescent staining of BMMs cultured on SLA surfaces for 24 h. DAPI, blue; CCR7 (M1 marker), red; CD163 (M2 marker), green. **C** Semiquantitative analysis of the mean immunofluorescence intensity of BMMs. **D** Western blotting of IRAK4, M1 Mø (iNOS, CCR7), M2 Mø (CD206, CD163) of BMMs after cultured on the SLA surfaces with the addition of Il-1β or IRAK4i. **E** Quantitative analysis of the relative level of proteins. **F** The transcriptional levels of inflammation-related and wound healing-related genes of BMMs were evaluated via RT-qPCR. *p < 0.05, **p < 0.01, ***p < 0.001, ****p < 0.0001

SLA surface implants are the most widely used dental implant in the clinic and were selected for subsequent experiments. To exclude the negative influence on the cell viability by itself, IRAK4i was added to the BMMs at a series of concentrations, and the results showed that the cell viability remained at a plateau until the concentration was up to 125 nM (Sup Fig. 1). When 50 nM IRAK4i was added, there was no statistical difference in OD values among all groups (Fig. 1A). Therefore, 50 nM IRAK4i was adopted in the following experiments.

Macrophage polarization markers were detected at gene and protein levels. As shown in Fig. 1B and Fig. 1C, in the presence of IL-1β, the fluorescence intensity of CCR7 increased, while that of CD163 decreased. In contrast, IRAK4i suppressed the up-regulation of CCR7 and relieved the inhibition of CD163 in the simulated inflammatory microenvironment. The WB results also confirmed that the level of iNOS and CCR7 increased with the addition of IL-1β, while that of CD163 and CD206 decreased (Fig. 1D and Fig. 1E). The expression of IRAK4 was effectively inhibited by the introduction of 50 nM IRAK4i (Fig. 1D).

To verify the functional status of macrophages, RT-qPCR was used to detect a series of inflammation-related genes and wound-healing genes (Fig. 1F). IL-1β-stimulated macrophages showed a sharply higher expression level of pro-inflammatory genes including *iNOS*, *Il-6*, and *Tnf-α*, and a lower expression level of tissue-healing genes such as *Tgf-β* and *Bmp2*. However, IRAK4i reversed this situation, significantly down-regulating the inflammation-related genes. The expression of *Vegf*, *Tgf-β,* and *Bmp2* slightly increased without statistical significance. Our results indicated that IL-1β promotes macrophages to polarize to the M1 phenotype and induces an inflammatory response, but IRAK4i impeded this inflammation and restored the polarization of M2 macrophages.

### IRAK4i prevented excessive osteoclastogenesis and relieved the inhibition of FBGCs formation on the SLA surface under simulated inflammatory condition in vitro

**Fig. 2:**
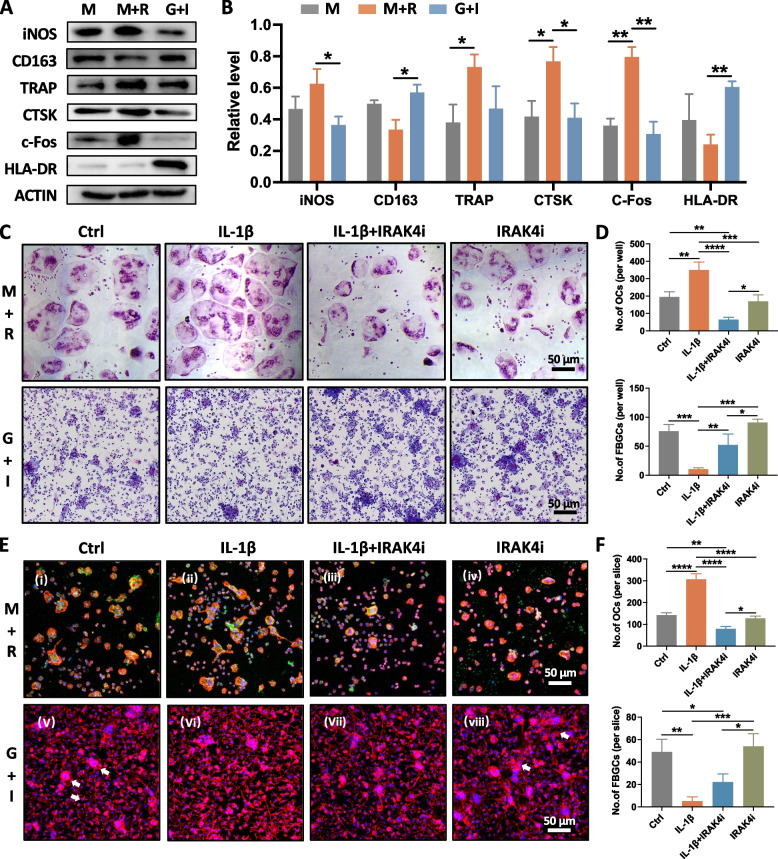
IRAK4i impeded excessive osteoclastogenesis and restored the formation of FBGCs on the SLA surface or on glass caused by inflammation in vitro. **A** Western blotting of iNOS, CD163, TRAP, CTSK, c-Fos, and HLA-DR of BMMs cultured on SLA surfaces in the presence of M-CSF (50 ng/ml) and RANKL (50 ng/ml), or GM-CSF (50 ng/ml) and IL-4 (50 ng/ml). **B** Quantitative analysis of the relative level of proteins in Fig. 2A. **C** Representative images of the formation of osteoclasts or FBGCs cultured on glass in the presence of M-CSF and RANKL, or GM-CSF and IL-4 were visualized by a microscope. Scale bar = 50 μm. **D** Semiquantitative analysis of the number of multinucleated cells per well (nuclei > 3) in Fig. 2C. **E** Representative images of the formation of osteoclasts or FBGCs cultured on SLA surfaces were visualized by immunofluorescence staining. DAPI, blue; F-actin, red; TRAP, green. Scale bar = 50 μm. **F** Semiquantitative analysis of the number of multinucleated cells per disk (nuclei > 3) in Fig. 2E. *p < 0.05, **p < 0.01, ***p < 0.001, ****p < 0.0001

BMMs were cultured, on the SLA surface or om glass in osteoclast-inductive medium with the addition of M-CSF (50 ng/ml) plus RANKL (50 ng/ml), or FBGC-inductive medium added with GM-CSF (50 ng/ml) plus IL-4 (50 ng/ml) until the multinucleated cells occurred. The expression of respective markers was detected by western blotting (Fig. 2A). And semiquantitative analysis showed that osteoclasts highly expressed iNOS, TRAP, CTSK, and c-Fos, while FBGCs highly expressed CD63 and HLA-DR (Fig. 2B).

The role of IRAK4i and its effect on osteoclast and FBGC formation on implant surfaces was then assessed. As shown in Fig. 2C, 2E, osteoclasts and FBGCs were both multinucleated with typical F-actin rings. Osteoclasts on the SLA surfaces displayed multi-cell nuclei, small cellular size, and homogeneous actin rings. The size of osteoclasts was obviously larger on the smooth glass surface than on the rough SLA surface, which indicated the effect of different surfaces on the morphology of osteoclasts. The morphology of FBGCs exhibited less difference on both surfaces. No matter on which surfaces, the quantitative analysis indicated that IL-1β increased the number of TRAP^+^ osteoclasts, but IRAK4i reversed the increment caused by IL-1β (Fig. 2D, 2F). Conversely, in the simulated inflammatory condition, the formation of FBGCs was sharply reduced, but IRAK4i treatment alleviated this inhibition. Compared with the control group, IRAK4i alone did not significantly influence the number of osteoclasts and FBGCs. From the observed phenomenon on the SLA titanium surface, we speculated that, maybe in the bone-implant interface in vivo, IRAK4 is a regulator between osteoclast and FBGC formation under inflammatory circumstances.

### Inflammation promoted the differentiation of osteoclasts, while IRAK4i restored the FBGC differentiation inhibited by inflammatory cytokines

**Fig. 3:**
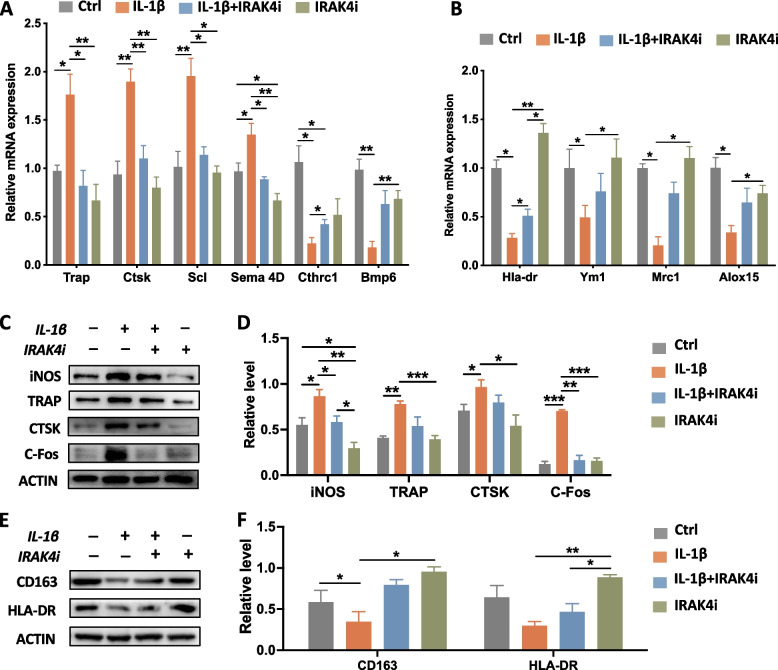
Differentiation of osteoclasts was promoted by IL-1β, while reversed by IRAK4i, and vice versa for FBGCs formation. **A** The transcriptional level of characteristic genes of osteoclasts with different treatments was analyzed. **B** The transcriptional level of functional genes of FBGCs with different treatments was analyzed. **C-D** Representative images and quantitative analysis of iNOS, TRAP, CTSK, and c-Fos in induced osteoclasts with different treatments. **E–F** Representative images and quantitative analysis of CD163 and HLA-DR in induced FBGCs with different treatments of four groups. *p < 0.05, ** p < 0.01, *** p < 0.001

The effect of IRAK4 on osteoclast or FBGC differentiation on SLA surfaces was further analyzed respectively. At the transcription level (Fig. 3A), besides *Trap* and *Ctsk*, bone resorption-related genes *sclerostin (Scl*) and *Sema 4D* of osteoclasts were significantly up-regulated in inflammatory conditions while such an up-regulation was impeded by IRAK4i. Bone formation-related genes *Cthrc1* and *Bmp6* were down-regulated by IL-1β and subsequently rescued by IRAK4i. Furthermore, the transcriptional levels of *Ym1, Mrc1* (M2 marker), and *Alox15* (an enzyme functioning in wound healing) were analyzed (Fig. 3B). Lower expression of *Ym1, Mrc1,* and *Alox15* genes was found in FBGCs under IL-1β stimulation, but their expression levels were reversely elevated by simultaneous IRAK4i treatment.

At the protein level (Fig. 3C), IRAK4i influenced the expression of polarization markers (iNOS and CD163) in osteoclasts in the same trend as that of BMMs. Osteoclast differentiation markers such as c-Fos underwent higher expression under inflammatory circumstances but were decreased after IRAK4i treatment (Fig. 3D). However, there was no statistical difference between TRAP and CTSK, which was inconsistent with the expression at the gene level, possibly due to the difference in transcription level and translation level at the time point of expression. As indicated by the expression levels of iNOS, CD163 and HLA-DR (Fig. 3E), IL-1β promoted the iNOS of the differentiating FBGCs. IRAK4i therapy reversed it and promoted the expression of more CD163 under an inflammatory microenvironment (Fig. 3F).

Our results suggested that on SLA titanium surfaces, IRAK4i treatment directly alleviates the IL-1β-driven inflammatory response not only in BMMs, but also in osteoclast and FBGC, both of which seems to be mediated by M1/M2 polarization. In addition, IRAK4 also could act as a differentiation switch in the regulation of osteoclasts and FBGCs.

### The osteogenic differentiation of BMSCs in conditioned media from osteoclasts or FBGCs inductively cultured on the SLA surfaces

**Fig. 4:**
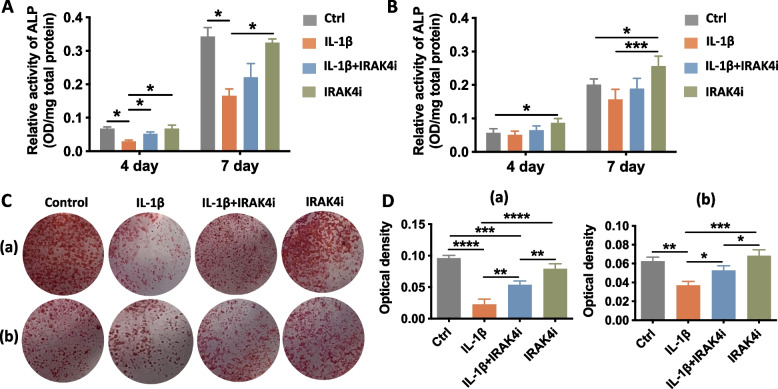
Anabolic effects of conditioned medium from osteoclasts and FBGCs influenced the osteogenic differentiation of BMSCs. BMSCs were treated with a mixture of an osteogenic medium (50% v/v) and various conditioned mediums from osteoclasts or FBGCs cultured on the SLA surfaces with or without IL-1β and IRAK4i. **A-B** After 4 days or 7 days cultured with the conditioned medium mixture from osteoclast or FBGC, ALP activity of BMSCs was respectively analyzed. **C-D** BMSCs were cultured in the conditioned medium mixture from osteoclasts (**a**) or FBGCs (**b**) for 21 days, respectively, then the mineralization nodules were stained with Alizarin Red S and quantified by cetylpyridinium chloride solution. *p < 0.05, **p < 0.01, ***p < 0.001, ****p < 0.0001

Considering the coupling signaling mechanism between multinucleated cells and BMSCs, we further investigated whether these subtypes of multinucleated cells and their shift during the inflammatory condition and IRAK4i treatment could modulate the osteogenic differentiation of BMSCs in a paracrine manner. The supernatant of osteoclast or FBGC cultures was collected respectively for indirect co-culture with BMSCs. According to the ALP activity analysis (Fig. 4A, 4B), BMSCs incubated with conditioned medium harvested from osteoclasts in the IL-1β group showed the lowest expression of ALP activity than the other groups on the 4th and 7th day. In the IL-1β + IRAK4i group, the ALP activity was significantly higher than that of the IL-1β group on the 4th day, which became less obvious on the 7th day. Compared with the control group, no significant difference was found in ALP activity of BMSCs incubated with conditioned medium from FBGCs in the IL-1β group and the IL-1β + IRAK4i group.

Concerning matrix calcification, the calcium nodule formation of BMSCs was analyzed by alizarin red staining (Fig. 4C). Quantitative analysis (Fig. 4D) showed BMSCs subjected to conditioned medium collected from osteoclasts in the IL-1β group displayed significantly less nodule formation than other groups, but it was reversely increased in the IL-1β + IRAK4i group. As for FBGCs conditioned medium, the IL-1β + IRAK4i group also showed increased formation of mineralized nodules than the IL-1β group. IRAK4i alone group exhibited the most mineralized nodule formation.

Taken together, it was indicated that both types of multinucleated cells may secrete coupling signals to affect the matrix calcification of BMSCs that was inhibited under an inflammation environment. IRAK4i treatment antagonized inflammatory osteogenesis inhibition indirectly via osteoclasts and FBGCs in a paracrine way.

### Effects of IRAK4i on macrophage polarization and multinucleated cells distribution in vivo

A transient acute inflammatory response occurs early in the establishment of osseointegration after implantation. Our *in-vitro* experiments provided a reasonable basis for predicting that IRAK4i may play a role in the initial stage of osseointegration, as to how to make the implant better through the early inflammatory stage and fasten osseointegration. IRAK4i was locally injected into the implant region to inhibit its expression using various methods (Sup Fig. 2). The most effective one was to inject IRAK4i (2500 nM, 100 μL) into the sub-periosteum of the implant site for three consecutive days prior to the implantation surgery.

**Fig. 5:**
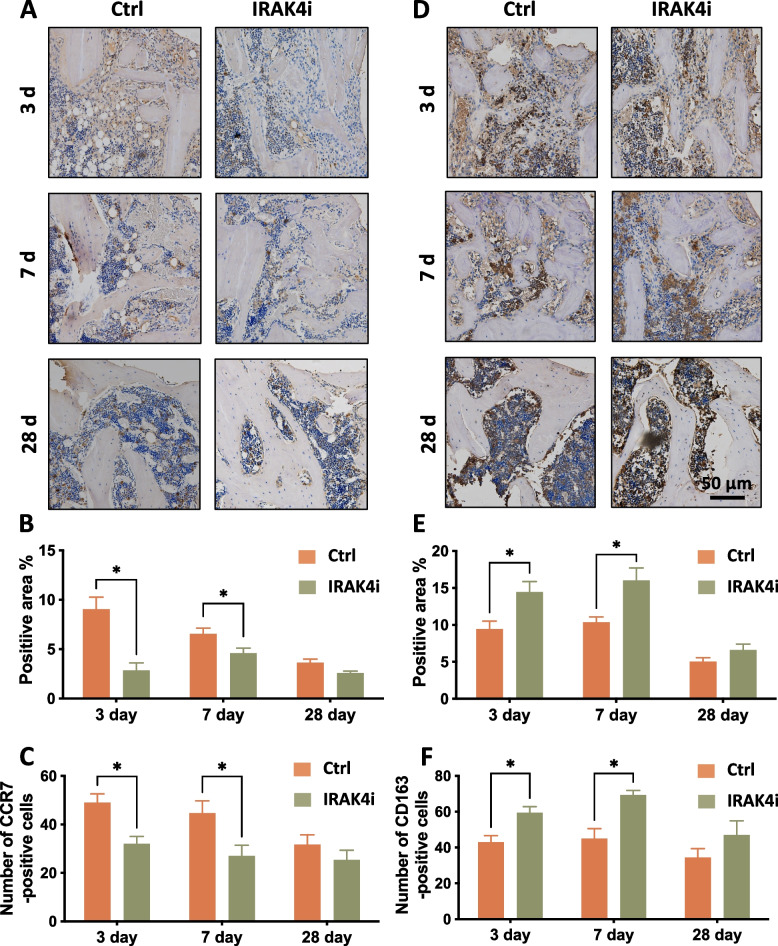
Effects of IRAK4i on macrophage polarization in vivo. SD rats were injected with PBS (Ctrl) or IRAK4i (2500 nM, 100 μL) subperiosteally at the implant sites for three consecutive days before implanted with SLA implants in the tibiae. **A**, **D** Immunohistochemistry staining of CCR7 (brown, M1 marker) (**A**) or CD163 (brown, M2 marker) (**D**) were done in the tissue surrounding implants at 3, 7, and 28 days after implantation. **B, E** Semiquantitative analysis of CCR7-positive area (**B**) or CD163-positive area (**E**) were done in the tissue surrounding implants. **C, F** Quantitative analysis of the number of CCR7^+^(**C**) and CD163^+^(**F**) cells on the bone-implant interface at each time point. *p < *0.05*

In Fig. 5A and 5D, CCR7 and CD163 were chosen as M1 and M2 phenotype markers, respectively. The number of M1 positive cells peaked earlier (on the 3rd day) than M2 positive cells (on the 7th day). Our result showed that the IRAK4i group decreased the positive area of M1 cells and increased that of M2 cells compared to the control group at the 3rd and 7th day after implantation (Fig. 5B, 5E). Further statistical analysis of the number of CCR7^+^ (Fig. 5C) and CD163^+^ (Fig. 5F) cells at the bone-implant interface showed that the trend was consistent with the results in the positive area percentage. It showed the effectiveness of IRAK4i therapy on the modulation of polarization of the monocyte-macrophage lineage cells in favor of bone tissue healing.

**Fig. 6:**
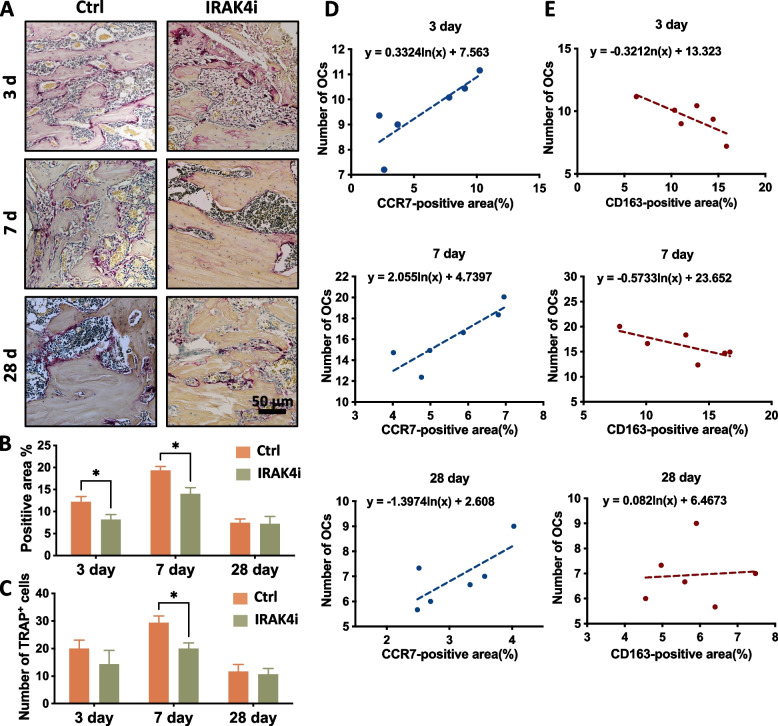
IRAK4i inhibited TRAP (osteoclast marker) expression levels in the peri-implant bone tissue. **A** Representative images of TRAP staining (pink) after 3, 7, and 28 days of implantation. **B** Semiquantitative analysis of TRAP^+^ cells at implantation sites at each time point. **C** Quantitative analysis of the number of TRAP^+^ cells on the bone-implant interface at each time point. **D, E** The correlation regression analysis of the number of osteoclasts and polarized macrophages displayed a linear correlation. The average number of osteoclasts in different groups positively correlated with the percentage of CCR7-positive area (**D**). While the average number of osteoclasts in different groups negatively correlated with the percentage of CD163-positive area (**E**). *p < 0.05

Regarding the formation of osteoclasts, TRAP staining was used in the same series of decalcified disks. TRAP^+^ cells with lacunose marginal folds were found on the new bone surfaces (Fig. 6A). On the 3rd and 7th days, the local administration of IRAK4i resulted in fewer TRAP^+^ osteoclasts, but there was no significant difference on the 28th day (Fig. 6B). Further, analysis of the number of TRAP-positive cells at the bone-implant interface showed that the fewer TRAP^+^ cells at the interface on the 7th day (Fig. 6C). In addition, HLA-DR was highly expressed in multinucleated cells, but not in osteoclasts. So, it serves as a marker to distinguish FBGCs from osteoclasts. In Sup Fig. 3, HLA-DR^+^ cells were observed around the bone tissue rather than attached to the surface of the bone. Specifically, FBGCs rarely formed within 3 days, but increased after 7 days and persisted after 28 days. However, there was no statistical difference between the control and the IRAK4i groups.

Based on the immunohistochemical analysis, we further analyzed the correlation between the number of osteoclasts and the percentage of M1 or M2 macrophage positive area. As shown in Fig. 6D and 6E, on the 3rd and 7th day, the number of osteoclasts was positively correlated with the CCR7-positive area and negatively correlated with the CD163-positive area, which was similar to the trend of *in-vitro* experiments. Due to the scarcity of FBGCs in the immunohistochemical disks, it is regretfully unable to interpret the relationship between FBGCs and macrophage polarization in vivo for quantitative analysis (Sup Fig. 3).

### Effects of IRAK4i treatment on early osseointegration in vivo

**Fig. 7:**
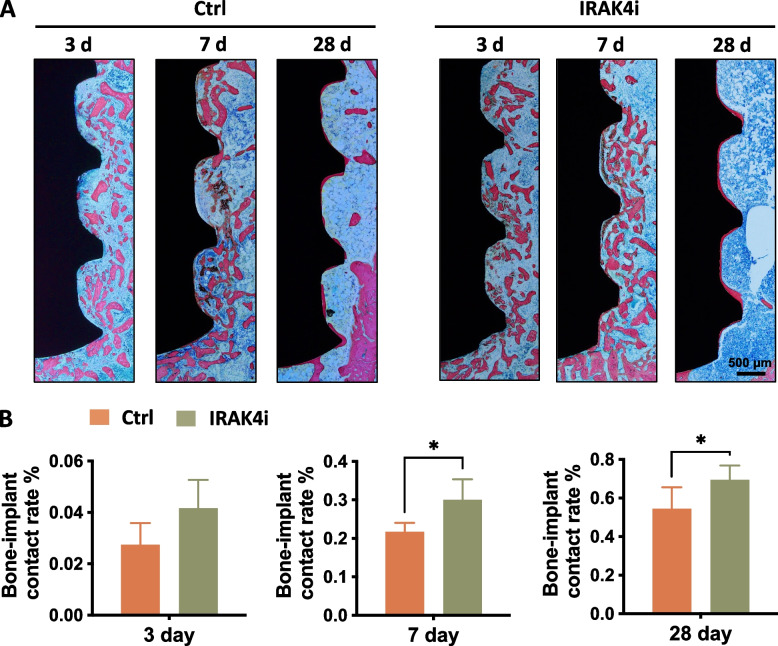
Histomorphometric evaluation of osseointegration of SLA implants untreated and pre-treated with IRAK4i after implantation for 3, 7, and 28 days. **A** Representative images of methylene blue/acid fuchsin staining showed the bone (pink) formed around the titanium implants (black) at each time point after implantation. **B** Histomorphometry of bone-implant contact ratio (BIC%) at 3, 7, 28 days. *p < 0.05

Undecalcified disks including bone tissue and the implant were prepared in the central part of the implant along its longitudinal axis (Fig. 7A). On the 3rd day, the bone-implant contact seemed spotted along the surface, but with the extension of time, the contact became continuous. At the 7th and 28th day of implantation, the percentage of bone-implant contact ratio (BIC%) in the IRAK4i group was higher than that of the control group (Fig. 7B). This demonstrated that IRAK4i treatment is an effective strategy to modulate the initial inflammatory stage after implant placement and to promote osseointegration.

Based on the *in-vivo* experiments, it was shown that IRAK4i can reduce the number of M1 macrophages and osteoclasts while increasing the number of M2 macrophages during the initial acute inflammatory stage of implant placement and shows a better osteointegration effect than the control group.

## Discussion

Biomaterial implantation and surgical injury inevitably lead to inflammation and foreign body reactions, followed by the formation of multinucleated cells, reaching an equilibrium under moderate immune response [2, 25]. Macrophage polarization, multinucleated cells formation, and subsequently, their interactions with BMSCs are critical to bone regeneration and functional integration of implants [26], but the underlying mechanisms remain unclear.

The current study suggests that IRAK4 may influence the formation of osteoclasts and FBGCs by regulating the polarization of macrophages, and ultimately play a role in osteointegration of implant. Under the inflammation circumstance in vitro and in vivo, IRAK4 inhibition can transform macrophage polarization from M1 to M2 phenotype, reduce the excessive osteoclastogenesis, and relieve the FBGC inhibition. It was indicated that the multinucleated cells indirectly took part in the osteogenic differentiation of BMSCs. The BIC% could also be enhanced by IRAK4i treatment in vivo. Collectively, IRAK4i was proved to be a promising target for immunomodulating peri-implant osseointegration via macrophage polarization and multinucleated cells transformation.

Macrophage fusion and multinucleation are fundamental processes during bone hemostasis around the bone-implant interface [27]. The formation of multinucleated cells and their role in osteointegration have been controversial. The mainstream view is that multinucleated cells are mainly osteoclasts and foreign body giant cells, among which osteoclasts are traditionally involved in the bone resorptive process [9, 14, 28]. However, studies have found osteoclasts are indispensable in bone remodeling after implantation. Osteoclasts contributed to the osteogenic differentiation of MSCs by secreting calcium ions, CTHRC1 and PDGF-B. Increased osteoclasts promoted the remodeling and resorption of new bone, leading to a coordinated replacement of biomaterials with mature bone [29]. Foreign body giant cells previously were the symbol of failure implantation, leading to fiber encapsulation. But recently, it has been found that they have a similar phenotype to M2-type macrophages and secrete some factors that promote tissue healing [30]. Polarization and fusion of macrophages regulate the formation of multinucleated cells and play different roles in the subsequent bone regeneration process.

IRAK4 is required for activation of the E3 ubiquitin ligase TNF receptor-associated factor 6 (TRAF6) by both MyD88 and TIR domain-containing adaptor protein (TRIF) [31]. MyD88 signaling regulates the network of the innate immune system, IL-1 ligand sensing, and osteoclast formation [19, 32]. IRAK4 is significantly up-regulated in the synovium of osteoarthritis, and Ad-shIRAK4 decreased its expression via inhibiting the TLR/IL-1R signaling pathway and alleviated synovitis and cartilage degeneration [33]. Moreover, IRAK4 kinase activity controls the activation of IRF5 which contributes to macrophage polarization. Antagonizing IRAK4 activity could partially abolish the nuclear translocation of IRF5 through down-regulating transcription of the gene associated with M1 phenotype-related genes (IL-12, IL-23, TNF, IL-6) and up-regulating that encoding IL-10 [34]. IRAK4i therapy markedly attenuated arthritis by inhibiting IRF5, M1 gene transcription, and Th1/Th17 cell differentiation [22].

A finding is that M1/M2 polarization and multinucleated cell differentiation are regulated under inflammatory conditions via IRAK4 on the titanium surfaces. IL-1β was used to mimic the inflammatory response, one highly expressed and driving cytokines in inflammation. Our results showed that the number of osteoclasts increased sharply in the presence of IL-1β, while the formation of FBGCs was inhibited. After IRAK4i treatment, excessive osteoclastogenesis and inhibition of FBGCs formation were rescued. More importantly, in the simulated inflammation circumstance in vitro and the early inflammatory stage after implant placement in vivo, inhibition of IRAK4 expression can transform macrophage polarization from M1 to M2 phenotype and reverse the modulation of osteoclasts and FBGCs, including their formation and biological activities. After pulling all the in vivo data together, the regression analysis displayed an average number of osteoclasts was positively correlated with the proportion of the M1 phenotype positive area and negatively correlated with the M2 phenotype positive area. The heterogeneity of multinucleated cells is comparable to macrophage polarization, and their M2 phenotype seems to be concerned with successful tissue remodeling. Collectively, IRAK4i was proved to be a promising approach for immunomodulating peri-implant osseointegration via M1/M2 polarization in the monocyte-macrophage lineage cells.

Despite the observation that implants induce the formation of multinucleated cells around the newly formed bone at the implant interface [35], the relationship between osteoclasts, FBGCs, and bone homeostasis has rarely been reported. It was demonstrated that the lack of multinucleated cells, such as osteoclasts and FBGCs stemming from the host response, would impede ectopic bone formation [36]. Considering that the foreign body response to an osteoinductive implant may determine its osteogenic potential, we hypothesize that the role of multinucleated cells stemming from the macrophage fusion would facilitate bone formation.

Osteoclasts play an anabolic role in bone remodeling. The conventional consensus is that osteoclasts mobilize chemotactic proteins from the matrix by bone resorption to recruit osteoblast precursors [37]. In other approaches, the bidirectional coupling between osteoclasts and osteoblasts via direct contact and the activation of osteoclasts promote osseous equilibrium and maturation of ossified bone in a paracrine mode, such as the secretion of chemotactic proteins [38, 39]. Despite bone resorption, non-absorbing osteoclasts lead to the osteogenic differentiation of osteoblastic cells in vitro and higher bone mass and strength in vivo, indicating that osteoclasts have anabolic functions during osseointegration and several "clastokines" could be released to regulate the behavior of osteoblastic cell [40, 41].

Although an excess of osteoclastogenesis will lead to an inhibited effect on osteogenesis due to Scl and Sema 4D [42, 43], CTHRC1 and BMP6 were confirmed to promote osteogenic differentiation [44, 45]. In this study, compared with foreign body giant cells, the conditioned medium from osteoclasts did promote osteogenic differentiation and mineralization of BMSCs. In the simulated inflammatory microenvironment, the anabolic effect from osteoclasts on BMSCs was inhibited, while down-regulation of IRAK4 reversed such a reaction. The gene expression of CTHRC1 and BMP6 significantly decreased under the inflammatory environment but reversely increased after IRAK4i treatment. The situation was vice versa for Scl and Sema 4D. The same western blotting showed that c-Fos might participate in the downstream signal pathway in this immunomodulation of osteoclastogenesis by IRAK4i.

FBGCs were previously thought to be characteristic cells of foreign body reaction that lead to fibrous encapsulation and implant failure [46]. Recent studies showed that the reduction of FBGC fusion after DC-STAMP knockout significantly down-regulated the differentiation of osteoblast, suggesting that FBGCs are closely involved in bone-biomaterial interactions [47]. These cells adhere to the biomaterial surface, form a special microenvironment between the cells and the surface, and release some degradable factors, such as active oxygen free radicals, degrading enzymes, and acids to increase the sensitivity of biomaterial degradation [48]. FBGCs could secrete certain anti-inflammatory factors and chemotactic signals such as IL-10, Cthrc1, Fizzl, and chemokines (MIP1, MCP1) to regulate the immune microenvironment and pre-osteoblasts [23, 49]. Although FBGCs did not directly promote osteoblastic differentiation of BMSCs, Ym1 and Alox15 were highly expressed in FBGCs, which play a role in wound healing and inflammation termination [50, 51].

There is still a limitation due to the conditioned mediums harvested from multinucleated cells mixed with polarized macrophages. The potential role of macrophages could not be excluded. Furthermore, considering the complexity of the bone osteoimmune microenvironment in vivo, the complete identification of multinucleated cells subtypes found in tissues and their characteristics are still unclear and controversial. It is promising to screen the synthetic signals of multinucleated cells and elucidate the underlying molecular mechanism.

## Conclusion

In this study, under inflammatory conditions, by transforming macrophages from M1 to M2, IRAK4i treatment could down-regulate the formation and activity of osteoclast and relieve the inhibition of FBGC generation, thus promoting osteogenic differentiation in BMSCs and improve the osteointegration. Our results may improve our understanding of the function of multinucleated giant cells and offer IRAK4i as a therapeutic strategy to improve implant osseointegration and help to eliminate the early implant failure. Further studies still need to investigate whether IRAK4i provides an effective approach to treating immune-inflammatory peri-implantitis and delayed implant failure after the establishment of implant osseointegration.

## Supplementary Information

**Additional file 1.**

## Data Availability

The datasets used and/or analyzed during the current study are available from the corresponding author on reasonable request.

## Abbreviations

IRAK4: Interleukin-1 receptor-activated kinase 4
FBGC: Foreign body giant cell
BMSC: Bone marrow stromal stem cell
BMM: Bone-marrow-derived macrophages
TLRs: Toll-like receptors
MIP-1α: Macrophage inflammatory protein-1α
IFN-γ: Interferon-γ
NF-κB: Nuclear factor kappa B
MAPK: Mitogen-activated protein kinase
IRF5: Interferon regulatory factor 5
SLA: Sand-blasted and acid-etched
M1: Classically activated macrophages
M2: Alternatively activated macrophages
iNOS: Inducible nitric oxide synthase
CCR7: C-C motif chemokine receptor 7
M-CSF: Macrophage colony stimulating factor 1
RANKL: Receptor ligand for NF-κB
GM-CSF: Granulocyte-macrophage colony-stimulating factor
IL-4: Interleukin-4
TRAP: Tartrate resistant acid phosphatase
CTSK: Cathepsin K
CM: Conditioned medium
OM: Osteogenic medium
ALP: Alkaline phosphatase
TNF-α: Tumor Necrosis Factor-α
TGF-β: Transforming Growth Factor β
BMP: Bone morphogenetic proteins
VEGF: Vascular endothelial growth factor
TRAF6: TNF receptor-associated factor 6
TRIF: TIR domain-containing adaptor protein
CTHRC1: Collagen triple helix repeat containing 1
DC-STAMP: Dendrocyte expressed seven transmembrane protein
