# Supplementary material for: IRAK4 inhibition: an effective strategy for immunomodulating peri-implant osseointegration via reciprocally-shifted polarization in the monocyte-macrophage lineage cells
Source: BMC Oral Health. 2023 May 8;23:265. doi: 10.1186/s12903-023-03011-0 (PMC10169473; doi:10.1186/s12903-023-03011-0)
Supplement: Supplementary file 1 — Additional file 1. [file 12903_2023_3011_MOESM1_ESM.docx]

**Supplementary material**

**Supplementary figure 1.** Cell viability assay of BMMs under various concentrations of IRAK4i (0, 15.625, 31.25, 62.5, 125, 250, 500 or 1000 nM).

**Supplementary figure 2.** (A) Western blotting was used to screen out the effective IRAK4i-introducing method among various administration alternatives *in vivo*. Western blot was performed on bone tissues from a distance of about 5 mm around the implants.

a1: Control-SLA group, SLA implant was injected with PBS (100 μL) at the same time of implantation.

a2: Soaking-SLA group, SLA implant was soaked in IRAK4i solution before implantation.

b1: Dropping-SLA group, SLA implant was dropped with IRAKi (2500 nM, 100 μL) at the same time of implantation.

b2: Dropping-polished titanium (PT, without screw) group, PT implant was dropped with IRAK4i (2500 nM, 100 μL) at the same time of implantation.

c1: 100 μL inhibitor-SLA group, SLA implant was inserted after injecting with IRAK4i (2500 nM, 100 μL) within bone marrow at the implant sites.

c2: 100 μL inhibitor-PT group, PT implant was inserted after injecting with IRAK4i (2500 nM, 100 μL) within bone marrow at the implant sites.

d1: Subperiosteal 100 μL inhibitor-SLA group, SLA implant was inserted after injecting with IRAK4i (2500 nM, 100 μL) subperiosteally at the implant sites for 3 consecutive days.

d2: Subperiosteal 100 μL inhibitor-PT group, PT implant was inserted after injecting with IRAK4i (2500 nM, 100 μL) subperiosteally at the implant sites for 3 consecutive days.

e1: Non-implant group.

(B) The original western blot images of IRAK4 and ACTIN.

**Supplementary figure 3.** HLA-DR immunostaining staining on the decalcified section of the control group (PBS) and IRAK4i group. Representative images of HLA-DR after 3 (A), 7 (B), 28 days (C) of implantation. Brown cells with more than three nuclei were regarded as FBGCs.

**Supplementary figure 4.** The original western blot images in full-length. (A) Western blotting of IRAK4, M1 Mø (iNOS, CCR7), M2 Mø (CD206, CD163) of BMMs after cultured on the SLA surfaces with the addition of Il-1β or IRAK4i. (B) Western blotting of iNOS, CD163, TRAP, CTSK, c-Fos, and HLA-DR of BMMs cultured on SLA surfaces in the presence of M-CSF (50 ng/ml) and RANKL (50 ng/ml), or GM-CSF (50 ng/ml) and IL-4 (50 ng/ml). (C) Western blotting of iNOS, TRAP, CTSK, and c-Fos in induced osteoclasts with different treatments. (D) Western blotting of CD163, and HLA-DR in induced FBGCs with different treatments of four groups.
